# Microstructure and Strengthening Effect of Coated Diamond Particles on the Porous Aluminum Composites

**DOI:** 10.3390/ma16083240

**Published:** 2023-04-20

**Authors:** Bisma Parveez, Nur Ayuni Jamal, Abdul Aabid, Muneer Baig

**Affiliations:** 1 Department of Manufacturing and Materials Engineering, Kulliyyah of Engineering, International Islamic University Malaysia, Kuala Lumpur 53100, Malaysia; 2 Department of Engineering Management, College of Engineering, Prince Sultan University, Riyadh 11586, Saudi Arabia

**Keywords:** porous Al composite, diamond particles, space holder technique, PMMA, compressive behavior

## Abstract

In this work, porous Al alloy-based composites with varying Ti-coated diamond contents (0, 4, 6, 12 and 15 wt.%) were prepared, employing the powder metallurgy route and using a fixed amount (25 wt.%) of polymethylmethacrylate (PMMA) as a space holder. The effects of the varying wt.% of diamond particles on the microstructure, porosities, densities and compressive behaviors were systematically evaluated. The microstructure study revealed that the porous composites exhibited a well-defined and uniform porous structure with good interfacial bonding between the Al alloy matrix and diamond particles. The porosities ranged from 18% to 35%, with an increase in the diamond content. The maximum value of plateau stress of 31.51 MPa and an energy absorption capacity of 7.46 MJ/m^3^ were acquired for a composite with 12 wt.% of Ti-coated diamond content; beyond this wt.%, the properties declined. Thus, the presence of diamond particles, especially in the cell walls of porous composites, strengthened their cell walls and improved their compressive properties.

## 1. Introduction

Porous materials are increasingly demanded materials for aerospace and automobile applications due to their structural and functional advantages. These materials possess high specific strength, high specific stiffness and outstanding energy absorption capabilities [1,2]. As a result, porous materials have received extensive interest. Among all metals, aluminum (Al) is the most commonly used for the development of porous materials due to its low weight-to-strength ratio [3,4]. However, during their development, it was found that their cell structure was irregular, and the cell size was non uniform as a result of their low viscous metal melts [5]. In addition, they also exhibited comparatively lower compressive strength [6,7], ultimately limiting their applications. Thus, to increase the viscosity of metal melts and enhance the strength of porous Al, the metal additives and reinforcement addition were considered as a good approach. Several researchers reported the influence of metal additives and reinforcement on both the foaming as well as compressive behaviors of the resultant composites. The introduction of the alloying elements such as tin (Sn), boron (B), copper (Cu), magnesium (Mg) or chromium (Cr) into the Al matrix has been found to enhance the properties of porous composites [8,9,10,11,12]. The effects of the addition of boron carbide (B_4_C), silicon carbide (SiC), alumina (Al_2_O_3_) and carbon nanotubes (CNTs) to Al or their alloys on the compressive properties have been extensively studied [13,14,15,16,17]. There are limited reports on the diamond particles in porous composites due to their lower chemical activity, making it difficult to achieve good interfacial bonding with the metal matrix during composite preparation [18]. Similarly, diamond has extremely low wettability with the Al matrix, resulting in poor interfacial bonding [19]. To overcome these problems, the surface modification of diamond particles was preferred, which involves depositing elements that have a strong tendency to form carbides such as titanium, tungsten, chromium or molybdenum on the surface of diamond particles [20,21,22]. The coating must react with the diamond and adhere well to matrix materials such as titanium (Ti), which allows a metallic layer to form directly from metallic Ti during the coating process, without any chemical reactions, where carbon combines with Ti much more easily (it has the highest negative free energy for carbide formation) [20,23]. Several researchers have worked on improving the interface bonding between diamond and the metallic matrix in recent years [24,25,26,27,28]. These composites combine the load bearing capacity of a ductile metal matrix with the high strength of diamond reinforcement [29]. Also, some hybrid composite materials were developed using additive manufacturing-3D printing process for high strength-to-weight ratio process with some other aspects of mechanical properties [30,31]. However, their extensive study is still required to explore their improved properties for applications as energy absorbers.

Additionally, in recent investigations for achieving porosities in composite foams fabricated by powder metallurgical method sodium chloride (NaCl), carbamide particles have been utilized [32,33,34,35,36]. These allow the porosities to be tailored, resulting in a porous structure with control over the shape, pore fraction and size [37]. However, there are some issues associated with them; some are toxic, while others do not completely leach out during the leaching process. Researchers have recently replaced them with PMMA particles due to their spherical shape, excellent formability and complete decomposition at a lower temperature range of 360 to 400 °C, leaving nearly zero residue behind [38,39]. Additionally, their spherical shape allows for control over the pore shape and pore size in the resultant porous composites, but there are inadequate reports on the porous composites fabricated using PMMA as space holders and using the powder metallurgy method [40,41]. Therefore, it is highly required to study the properties of porous composites fabricated using PMMA particles as space holders to explore their development and wide application.

In the present work, porous Al composites were manufactured by powder metallurgical methods using PMMA as space holders. The microstructure and pore morphology of fabricated composites using SEM and XRD were characterized. Finally, the effects of the Ti-coated diamond content on the porosity, density and compressive behavior were analyzed.

## 2. Materials and Method

### 2.1. Materials

The powders of magnesium (Mg), tin (Sn), copper (Cu) and boron (B) were mixed with aluminum (Al) powder, as per the wt.% shown in Table 1, to form an alloy matrix mix. These powders were procured from Nova Scientific Resources Sdn Bhd, Malaysia, and their addition was meant to facilitate liquid sintering and disrupt the oxide layer present on the surface of the Al particles. Further, titanium-coated diamond particles of an average particle size (approximately 45 μm) at varying contents (0, 4, 8, 12 and 15% by weight) were used as reinforcements. PMMA particles obtained from Sigma Aldrich Malaysia were employed as space holders at a fixed amount of 25 wt.% to achieve the controlled porosity.

### 2.2. Preparation of Diamond-Reinforced Porous Al Composites

The powder metallurgy technique was used to develop porous composites. It involved a mixing, compaction, and sintering process. The mixing process was accomplished in three steps. First, the powders of Al, Mg, Sn, Cu and B were mixed for 24 h at 300 rpm using a horizontal ball mill. The powder-to-ball ratio was set at one-tenth. Further, the alloy matrix mix was mixed with the Ti-coated diamond particles using an oscillatory mixer for 2 h at 800 rpm, followed by mixing the entire mixture with PMMA particles using an oscillatory mixer for 2 h at 800 rpm in the last step. Then, the compaction process was carried out by compressing the powder mix in a cylindrical die with a 10 mm diameter using a hydraulic press at 350 MPa pressure to obtain the compacts. The compacted specimens were then heat-treated at 450 °C for 1 h to remove the PMMA particles, followed by being sintered at 590 °C for 1.5 h in an argon atmosphere in a carbolite tube furnace.

### 2.3. Characterization and Testing

The morphology of the starting powder (Al, Mg, Sn, Cu, B, Ti-coated diamond, PMMA particles and composite powder mixture) and the microstructure of the developed porous composites were examined by using a scanning electron microscope (SEM) (JEOL JSM-6300F). The porosity and density were measured via the Archimedes principle. The relative foam density of the samples was measured as per ASTM D3575 by using the equation given below.

(1) $$\mathrm{Relative}\;\mathrm{density}=\frac{P_{s}(g/{cm}^{3})}{S_{d}(g/cm{}^{3})}$$

where *P_s_* is the density of the porous Al composite sample in terms of g/cm^3^, and *S_d_* is the total density of solid raw materials (2.7 g/cm^3^).

To identify phase transformations, the X-ray diffraction patterns of sintered porous Al composites were acquired using (XRD, PAN analytical empyrean 1032) and Cu K radiation. The XRD patterns were achieved in the range of 20° to 80°. The compression testing was carried out using a uniaxial compression testing machine (Dartec model 3500 universal testing machine) at a constant crosshead speed of 0.5 mm/min and a load cell of 30 kN. The energy absorption capacity (W) of the resulting porous composites was calculated using the stress–strain curve and the following equation [42].

(2) $$W=\int_{0}^{\varepsilon }\sigma \;d\varepsilon$$

where *σ* and ε are the compressive stress and strain, respectively.

## 3. Results and Discussion

### 3.1. Microstructural Analysis

**Figure 1:**
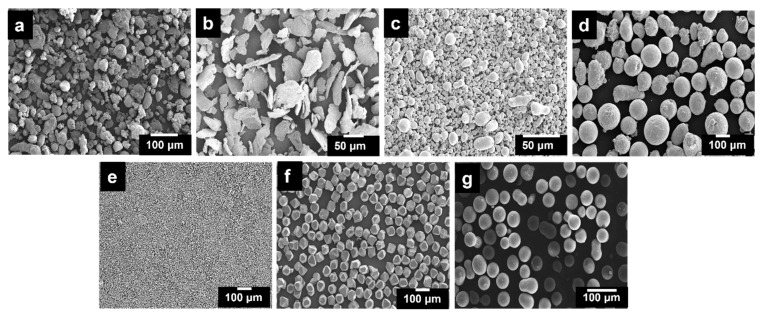
SEM micrography of (**a**) Al powder, (**b**) Mg powder, (**c**) Sn powder, (**d**) Cu powder, (**e**) Boron powder, (**f**) Ti-coated diamond particles and (**g**) PMMA particles.

Figure 1a–e show the SEM images of the starting powder, including Al, Mg, Sn, B, PMMA and Ti-coated diamond particles. Al, Sn, Cu, B and PMMA exhibited a spherical shape, as evident in Figure 1a–g. In contrast, Mg and the diamond particles were irregularly shaped, as shown in Figure 1b–e. Sn was incorporated in the Al matrix to enhance the fluidity of the porous Al composite during the sintering process. Additionally, to improve the sintering properties of Al, it is critical to break down the stable oxide film that has formed on the surface of the Al particles. For this purpose, Mg was added to the Al matrix [43]. Further, Cu and B were added to improve the strength of the Al matrix [10,44,45]. Additionally, these elements impact the various characteristics of molten Al, such as the melting point during the formation of a foam structure, the surface tension, and the viscosity. The melting point and the surface tension greatly influence the relative density and the cell size of Al foams made with molten alloys [46,47].

**Figure 2:**
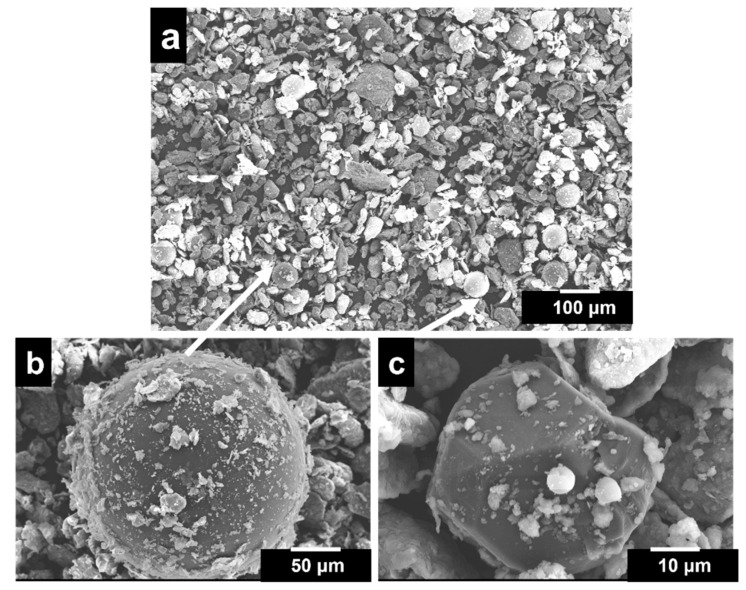
SEM micrograph of (**a**) elemental powder mixture, (**b**) PMMA particle and (**c**) Ti-coated diamond particle.

Further the three-step mixing resulted in regular and lamellar particle shapes of powders, structured with a smaller particle size (average particle size of 20 μm), as illustrated in Figure 2a. Mixing the powder for a long time resulted in the strain hardening of the powder particles. This results in brittleness, causing the fragmentation and formation of more equiaxed and finer particles [48]. Additionally, mixing PMMA particles with the binder prior to mixing with the metallic mix results in the sticking of metallic powders to the PMMA surface, as shown in Figure 2b. Additionally, metallic particles adhere to the surface of diamond particles, as shown in Figure 2c.

**Figure 3:**
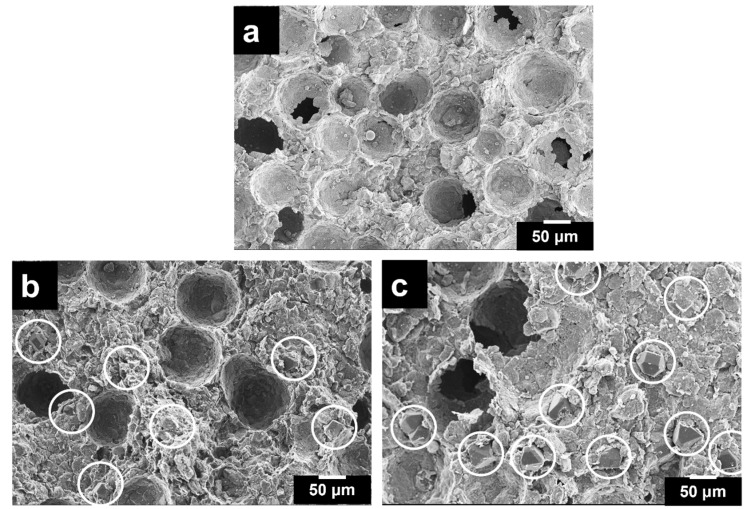
SEM micrographs of porous Al composites with (**a**) 0, (**b**) 12 and (**c**) 15 wt.% of diamond particles.

Finally, the SEM micrographs of the porous Al composites, as shown in Figure 3, revealed the closed macro-porous structure with an average macro-pore size ranging from 160 μm to 175 μm, which resembles the morphology of the as-received PMMA particle size and shape. These macro-pores are distributed uniformly and are separated from each other by a unique cell wall. It is vital to obtain pores that imitate the morphology of the starting space holder material, indicating that the pore structure can be tailored, depending on the space holder shape, size and content. Similar observations were made in steel foams developed using carbamide particles as space holders [49]. Moreover, the spherical-shaped pores have fewer edges and corners, thus reducing the surface roughness and decreasing the local stress concentrations and the inconsistent deformation during compression. This enhances the strength of the porous Al composite [50].

Additionally, Figure 3 reveals a well-defined shape and uniform distribution of pores in the case of composites with 0 and 12 wt.% of diamond particles as compared to 15 wt.% of diamond particles, which reveals the presence of distorted pores. These distorted pores are due to the agglomeration of diamond particles compressing the PMMA particles during compaction, distorting their shape [27]. Moreover, the micro porosities exhibited by the porous composites are mainly due to the insufficient availability of the matrix required to fill the gaps or pores between diamond particles [51]. As evident from the porosity and relative density values of porous composites shown in Table 2, the porosity increased from 18% to 35%, whereas their relative density decreased from 71% to 60% with an increase in the diamond content. Similar results were acquired in one of our recent works [52].

**Figure 4:**
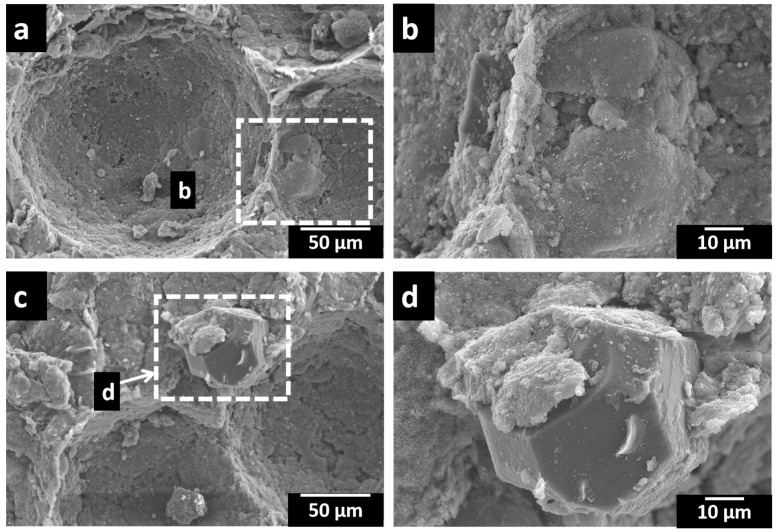
SEM micrographs of the cell wall of porous Al composites with (**a**,**b**) 12 wt.% and (**c**,**d**) 15 wt.% of diamond particles.

A remarkable improvement in the wettability of the Al alloy matrix and Ti-coated diamond particles was also observed, and these diamond particles were mostly found in the cell walls of the porous composites, as evident in Figure 4a,d. The well-bonded diamond particles in cell walls contribute to the enhancement of the cell wall strength, especially up to a 12 wt.% diamond particle content. A significant change in the porosity level and relative density for a 12 wt.% diamond content can be observed in Table 2. However, beyond this, the diamond particles bonding in the cell walls of porous composites reduces with the further increase in the diamond particle content due to the unavailability of a sufficient liquid matrix for wetting diamond particles. Thus, the higher wt.% of diamond composites exhibited lower relative densities and higher porosities due to the presence of cracks and voids in the interfaces of Al and diamond particles. A similar effect was reported by the researchers using diamond or CNT reinforcements in the Al matrix [21,53]. Further, this was found to impair the strength of the porous Al composites [54].

**Figure 5:**
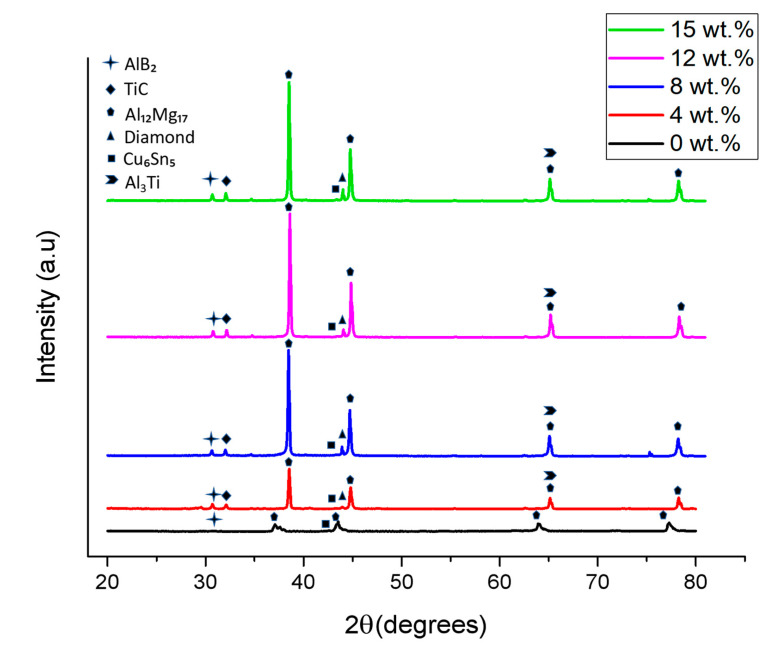
XRD analysis of porous Al composites at varying Ti-coated diamond particle contents.

The bonding can be attributed to the presence of sintering additives, and these additives form low-temperature intermetallic phases. The presence of such phases was revealed by the XRD analysis in Figure 5, the intermetallic Al-rich phases represented by the (111), (200), (220) and (311) diffraction peaks at 2 θ of 38.54°, 44.78°, 65.11° and 78.28°, respectively, and the Al_12_Mg_17,_ Cu_5_Sn_6_, AlB_2_ and Al_3_Ti phases in the porous Al composites. These phases are formed during sintering because of a partial reaction between constituents. Upon the addition of Sn and Cu to Al, the solid solubility of Sn in Cu occurs, thus producing Cu_5_Sn_6_ phases. As copper has higher affinity for Sn, Sn melts first and forms Cu-Sn phases in the vicinity of Cu [55]. This solid–solid transformation occurs around the temperature of 250 °C [56]. Further, when Mg is added to an Al matrix, the Al primarily precipitates along a grain boundary in the form of the Al_12_Mg_17_ phase at temperatures below 430 °C [57]. Additionally, boron tends to enrich at the interface due to the pull of chemical bond forces. Finally, the interface-enriched B reacts spontaneously with the Al alloy matrix around 590 °C to form AlB_2_ [58,59]. The formation of an intermetallic is found to improve the properties [60]. 

Additionally, due to the presence of Ti coating on diamond particles, during the coating process, the carbon atoms of diamond diffuse into the Ti-coated layer, occupying the octahedral interstitial positions of its lattices in Ti crystal-forming δ-TiC. This δ-TiC transition layer combines metallurgically with diamond, and the outer α-Ti layer remains on the outer surface. Upon the addition of Ti-coated diamond to the Al alloy matrix, the outer α-Ti later enables the wetting of the Al alloy matrix [61]. As a result, the Al_3_Ti phase was formed around the temperature of 590 °C [62,63], as evident from the XRD peaks at 38.46° and 64.56°, as shown in Figure 5; similar peaks were observed in other research works [58]. Additionally, an improvement in the peak reflection of the Al_12_Mg_17_ can be seen in Figure 5, indicating that either the amount of (Al_12_Mg_17_) increases or the crystallinity of the (Al_12_Mg_17_) phase increases with the increase in the Ti-coated diamond content.

### 3.2. Compressive Behavior

**Figure 6:**
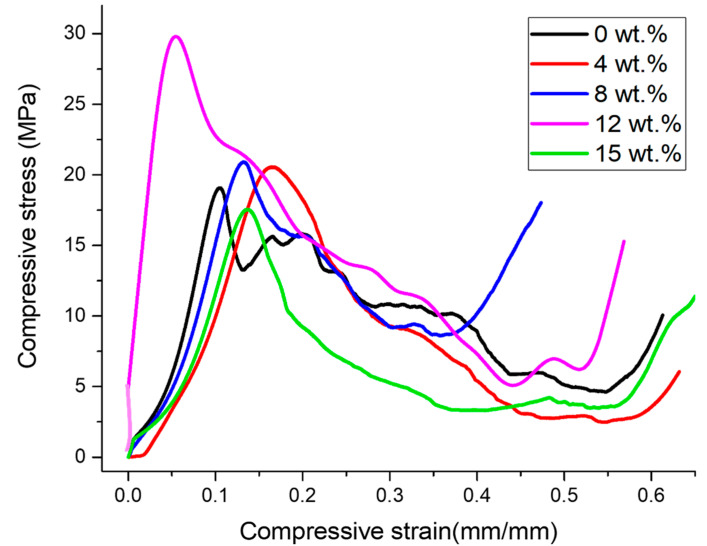
Stress–strain curve for varying diamond particle contents in the porous Al composite.

The porous Al composites under compression revealed an accurate classical deformation pattern, which can be classified into three phases: (a) The initial phase, known as the linear elastic region, where cell wall bending and face stretching take place; (b) The second phase, called the plateau region, where plastic deformation occurs at constant flow stress; and (c) The third phase, also called the densification region, characterized by a region of a sudden increase in flow stress [64,65], as evident in Figure 6. Also in Figure 6, it can be observed that there was an improvement in the plateau stress of porous Al composites upon the addition of Ti-coated diamond as a reinforcement. This can be attributed to the presence of harder reinforcement in the cell walls of the Al alloy matrix, which strengthens the Al alloy matrix by the Orowan mechanism, thus increasing their plateau stress. In addition, the increase in compressive properties is due to the solid solution strengthening that occurs as a result of the addition of alloying elements in the Al matrix [66]. However, it increased up to 12 wt.%; beyond this, the strength declines, mainly due to the presence of higher porosities, making elastic deformation easier. Additionally, this is due to the cell wall brittleness resulting from the weak adhesion force between the Al alloy matrix and diamond reinforcement as a result of the availability of the insufficient matrix for wetting diamond particles. Consequently, the porous specimen is unable to sustain the load applied and causes the collapse of the cell walls at the weakest point with the lowest density, a high stress distribution and the initiation of cracks, thereby reducing the plateau stress considerably [16].

The porous Al composite with (12 wt.%) diamond exhibited the highest value of plateau stress, as shown in Table 3. This is due to the presence of denser cell walls due to the presence of well-bonded diamond particles in cell walls, as evident in Figure 4, resulting in higher bending and buckling deformation resistance. The same behavior was observed in CNT-reinforced Al matrix foam [67]. In addition, the crystallinity of intermetallic phases was found to increase (Figure 5) with an increase in the Ti-coated diamond content, thereby assisting in the improvement of the compressive properties.

Further, from the area under stress–strain curves shown in Figure 5, the energy absorption capacity of the porous composites was measured. As evident in Figure 5, the area under stress–strain curve is larger for the porous Al composites with a 12 wt.% diamond content and was recorded to be 7.46 MJ/m³ as compared to the previous study, where the energy absorption capacity was reported to be 1.41 MJ/m³ in the resultant porous Al (30 wt.% of PMMA) without diamond particle reinforcement [64]. Additionally, due to the presence of good interfacial bonding between the diamond and Al alloy matrix as a result of Ti-coating, there occurs load transfer between them, resulting in stress concentration, thereby leading to improved strength. With the increase in the diamond content, the compressive strain remains nearly constant; however, the compressive stress levels decrease, thereby decreasing the energy absorption capacity. This is probably due to the presence of a more homogeneous pore structure of porous composites up to 12 wt.%, as shown in Figure 3, thus demonstrating better compressive strength and energy absorption capacity. However, beyond this wt.%, the weak bonding of the diamond particle in the Al alloy matrix leads to poor energy absorption capacity as a result of weak cell structures that can hardly bear higher compressive loading prior to fractures [17]. Therefore, this elucidates that the introduction of diamond as a reinforcement can effectively increase the compressive properties of the porous Al composites.

## 4. Conclusions

Porous Al composites with varying Ti-coated diamond particle contents (0, 4, 8, 12 and 15 wt.%) and PMMA (25 wt.%) as a space holder were successfully developed using the powder metallurgy technique. The key findings of this study are summarized as follows:

The uniform distribution of particles was obtained after a three-step mixing process, and the resultant composite mix consisted of a regular and lamellar structure with a smaller particle size.

(1) The microstructure of the porous Al composites revealed a uniformly distributed porous structure with less formation of micro-pores and cracks. The pore morphology resembled that of space holders and can thus be controlled. The spherical porosities improve the properties. The porous composites up to 12 wt.% exhibited a homogeneous distribution of pores and diamond particles.
(2) The porosity and relative densities were found to be maximum for porous Al composites with a 12 wt.% diamond content due to the better interfacial bonding between the Al alloy matrix and diamond particles as a result of the intermetallic and Ti-coating on diamond particles.
(3) The maximum plateau stress and energy absorption capacity were found to depend on the relative density, and their values were 31.51 MPa and 7.46 MJ/m^3^, respectively, for 12 wt.% diamond.
(4) Therefore, better microstructural and compressive behavior was obtained for porous Al composites with 12 wt.% of diamond particles; beyond this, the agglomeration of diamond particles occurred.

## Figures and Tables

**Table 1:** Particle size and purity of starting materials.

Constituents	Material	Average Particle Size (µm)	Purity (%)	Wt.%
	Al	45	99.9	94
	Mg	10	99.9	1
Alloy Matrix	Sn	45	99.5	2
	Cu	50	99.5	2
	B	10	99.5	1
Reinforcement	Ti-coated diamond	45	99.5	0, 4, 8, 12 and 15
Space holder	PMMA	150	99.9	25

**Table 2:** Porosity and relative density of porous Al composites.

wt.%	Porosity (%)	Relative Density (%)
0	18	0.71
4	20	0.65
8	24	0.63
12	26	0.69
15	35	0.60

**Table 3:** Compressive properties of porous Al composites.

wt.%	Plateau Stress (MPa)	Energy Absorption Capacity (Mj/m^3^)
0	19.08	4.25
4	20.50	4.69
8	20.86	5.43
12	31.51	7.46
15	17.56	3.54

## Data Availability

Not applicable.

## References

[1] Parveez B., Jamal N.A., Maleque A., Yusof F., Jamadon N.H., Adzila S. (2021). Review on advances in porous Al composites and the possible way forward. J. Mater. Res. Technol..

[2] Parveez B., Jamal N.A., Anuar H., Ahmad Y., Aabid A., Baig M. (2022). Microstructure and Mechanical Properties of Metal Foams Fabricated via Melt Foaming and Powder Metallurgy Technique: A Review. Materials.

[3] Albayrak O., Ipekoglu M., Altintas S. (2013). Effects of alumina (Al_2_O_3_) addition on the cell structure and mechanical properties of 6061 foams. J. Mater. Res..

[4] Almajid A.A. (2021). High-temperature deformation of naturally aged 7010 aluminum alloy. Metals.

[5] Attia M.S., Meguid S.A., Tan K.T., Yeo S.C. (2010). Influence of cellular imperfections on mechanical response of metallic foams. Int. J. Crashworthiness.

[6] Mu Y., Yao G., Liang L., Luo H., Zu G. (2010). Deformation mechanisms of closed-cell aluminum foam in compression. Scr. Mater..

[7] Said M.R., Tan C.F. (2008). The response of aluminium foams under quasi-static loading. Chiang Mai J. Sci..

[8] Hamdi A.A. (2018). Effect Of Magnesium and Tin As Sintering Additives on Microstructure and Compressive Properties of Porous Aluminum. Master’s Thesis.

[9] Sercombe T.B. (1998). Non-Conventional Sintered Aluminium Powder Alloys. Ph.D. Thesis.

[10] Ali S., Rani A.M.A., Altaf K., Baig Z. (2018). Investigation of Boron addition and compaction pressure on the compactibility, densification and microhardness of 316L Stainless Steel. IOP Conf. Ser. Mater. Sci. Eng..

[11] Sánchez de la Muela A.M., García Cambronero L.E., Malheiros L.F., Ruiz-Román J.M. (2022). New Aluminum Syntactic Foam: Synthesis and Mechanical Characterization. Materials.

[12] Li Y.G., Wei Y.H., Hou L.F., Guo C.L., Yang S.Q. (2015). Fabrication and compressive behaviour of an aluminium foam composite. J. Alloys Compd..

[13] Yang K., Yang X., He C., Liu E., Shi C., Ma L., Li Q., Li J., Zhao N. (2017). Damping characteristics of Al matrix composite foams reinforced by in-situ grown carbon nanotubes. Mater. Lett..

[14] Yang X., Hu Q., Li W., Song H., Zou T., Zong R., Sha J., He C., Zhao N. (2020). Compression-compression fatigue performance of aluminium matrix composite foams reinforced by carbon nanotubes. Fatigue Fract. Eng. Mater. Struct..

[15] Wang J., Yang X., Zhang M., Li J., Shi C., Zhao N., Zou T. (2015). A novel approach to obtain in-situ growth carbon nanotube reinforced aluminum foams with enhanced properties. Mater. Lett..

[16] Alizadeh M., Mirzaei-Aliabadi M. (2012). Compressive properties and energy absorption behavior of Al-Al2O3 composite foam synthesized by space-holder technique. Mater. Des..

[17] Chung C.Y., Chu C.H., Lee M.T., Lin C.M., Lin S.J. (2014). Effect of titanium addition on the thermal properties of diamond/Cu-Ti composites fabricated by pressureless liquid-phase sintering technique. Sci. World J..

[18] Wang L., Li J., Che Z., Wang X., Zhang H., Wang J., Kim M.J. (2018). Combining Cr pre-coating and Cr alloying to improve the thermal conductivity of diamond particles reinforced Cu matrix composites. J. Alloys Compd..

[19] Che Z., Li J., Wang L., Qi Y., Zhang Y., Zhang H., Wang X., Wang J., Kim M.J. (2016). Effect of diamond surface chemistry and structure on the interfacial microstructure and properties of Al/diamond composites. RSC Adv..

[20] Yang B., Yu J.K., Chen C. (2009). Microstructure and thermal expansion of Ti coated diamond/Al composites. Trans. Nonferrous Met. Soc. China.

[21] Zhang C., Cai Z., Wang R., Peng C., Qiu K., Wang N. (2016). Microstructure and thermal properties of Al/W-coated diamond composites prepared by powder metallurgy. Mater. Des..

[22] Ma S., Zhao N., Shi C., Liu E., He C., He F., Ma L. (2017). Mo 2 C Coating on Diamond: Different Effects on Thermal Conductivity of Diamond/Al and Diamond/Cu Composites.

[23] Suleiman M.J., Ahmad M.A., Mohamed N., Mohamad Junus Y., Ibrahim R., Mohamad M., Abdul Kadir M.R., Abu Kassim N.H., Awang R., Muhammad S. (2013). Effect of Atmosphere on the Sintered Titanium Alloy Produced By Metal Injection Molding (Mim) Technique. J. Ind. Technol..

[24] Dong Y., Zhang R., He X., Ye Z., Qu X. (2012). Fabrication, and infiltration kinetics analysis of Ti-coated diamond/copper composites with near-net-shape by pressureless infiltration. Mater. Sci. Eng. B Solid-State Mater. Adv. Technol..

[25] Zhang C., Cai Z., Wang R., Peng C., Feng Y. (2017). Enhancing densification capacity and properties of Al/diamond composites by partial liquid hot pressing. Surf. Coat. Technol..

[26] Abyzov A.M., Kruszewski M.J., Ciupiński Ł., Mazurkiewicz M., Michalski A., Kurzydłowski K.J. (2015). Diamond-tungsten based coating-copper composites with high thermal conductivity produced by Pulse Plasma Sintering. Mater. Des..

[27] Parveez B., Jamal N.A., Aabid A., Baig M., Yusof F. (2023). Experimental Analysis and Parametric Optimization on Compressive Properties of Diamond-Reinforced Porous Al Composites. Materials.

[28] Kovarik O., Cizek J., Yin S., Lupoi R., Janovska M., Cech J., Capek J., Siegl J., Chraska T. (2022). Mechanical and Fatigue Properties of Diamond-Reinforced Cu and Al Metal Matrix Composites Prepared by Cold Spray. J. Therm. Spray Technol..

[29] Sun Y., Zhang C., He L., Meng Q., Liu B.C., Gao K., Wu J. (2018). Enhanced bending strength and thermal conductivity in diamond/Al composites with B4C coating. Sci. Rep..

[30] Almajid A., Walter R., Kroos T., Junaidi H., Gurka M., Khalil K.A. (2021). Development of polypropylene/polyethylene terephthalate microfibrillar composites filament to support waste management. Polymers.

[31] Almajid A., Walter R., Kroos T., Junaedi H., Gurka M., Khalil K.A. (2021). The multiple uses of polypropylene/polyethylene terephthalate microfibrillar composite structures to support waste management—composite processing and properties. Polymers.

[32] Aboraia M., Sharkawi R., Doheim M.A. (2011). Production of aluminium foam and the effect of calcium carbonate as a foaming agent. J. Eng. Sci..

[33] Nakamura T., Gnyloskurenko S.V., Sakamoto K., Byakova A.V., Ishikawa R. (2002). Development of new foaming agent for metal foam. Mater. Trans..

[34] Mukherjee M., García-Moreno F., Jiménez C., Rack A., Banhart J. (2017). Microporosity in aluminium foams. Acta Mater..

[35] Jiang B., Wang Z., Zhao N. (2007). Effect of pore size and relative density on the mechanical properties of open cell aluminum foams. Scr. Mater..

[36] Mohd Razali R.N., Abdullah B., Ismail M.H., Ahmad U.K., Idham M.F., Rasmli A. (2013). Mechanical properties of aluminium foam by conventional casting combined with NaCL space holder. Appl. Mech. Mater..

[37] Krommenhoek M., Shamma M., Morsi K. (2017). Processing, characterization, and properties of aluminum–carbon nanotube open-cell foams. J. Mater. Sci..

[38] Jamal N.A., Maizatul O., Anuar H., Yusof F., Nor Y.A., Khalid K., Zakaria M.N. (2018). Preliminary development of porous aluminum via powder metallurgy technique. Mater. Werkstofftech.

[39] Tan P.P., Mohamad H., Anasyida A.S. (2018). Properties of Porous Magnesium Using Polymethyl Methacrylate (PMMA) as a Space Holder. J. Phys. Conf. Ser..

[40] Li B.Q., Wang C.Y., Lu X. (2013). Effect of pore structure on the compressive property of porous Ti produced by powder metallurgy technique. Mater. Des..

[41] Bi Y., Zheng Y., Li Y. (2015). Microstructure and mechanical properties of sintered porous magnesium using polymethyl methacrylate as the space holder. Mater. Lett..

[42] Baumeister J., Banhart J., Weber M. (1997). Aluminium foams for transport industry. Mater. Des..

[43] Showaiter N., Youseffi M. (2008). Compaction, sintering and mechanical properties of elemental 6061 Al powder with and without sintering aids. Mater. Des..

[44] Kang B., Kong T., Dan N.H., Phuong D.D., Ryu H.J., Hong S.H. (2021). Effect of boron addition to microstructure and mechanical properties of refractory Al 0.1 CrNbVMo high-entropy alloy. Int. J. Refract. Met. Hard Mater..

[45] Dixit M., Srivastava R. (2019). The effect of copper granules on interfacial bonding and properties of the copper-graphite composite prepared by flake powder metallurgy. Adv. Powder Technol..

[46] Miyoshi T., Hara S., Mukai T., Higashi K. (2001). Development of a closed cell aluminum alloy foam with enhancement of the compressive strength. Mater. Trans..

[47] German R.M., Rabin B.H. (1985). Enhanced sintering through second phase additions. Powder Metall..

[48] Hassani A., Bagherpour E., Qods F. (2014). Influence of pores on workability of porous Al/SiC composites fabricated through powder metallurgy + mechanical alloying. J. Alloys Compd..

[49] Bekoz N., Oktay E. (2012). Effects of carbamide shape and content on processing and properties of steel foams. J. Mater. Process. Technol..

[50] Yang K., Yang X., Liu E., Shi C., Ma L., He C., Li Q., Li J., Zhao N. (2017). Elevated temperature compressive properties and energy absorption response of in-situ grown CNT- reinforced Al composite foams. Mater. Sci. Eng. A.

[51] Mizuuchi K., Inoue K., Agari Y., Morisada Y., Sugioka M., Tanaka M., Takeuchi T., Tani J.I., Kawahara M., Makino Y. (2011). Processing of diamond particle dispersed aluminum matrix composites in continuous solid-liquid co-existent state by SPS and their thermal properties. Compos. Part B Eng..

[52] Parveez B., Jamal N.A., Raeez M. (2023). Investigation of Morphology and Compressive Properties of Diamond Reinforced Porous Aluminium Composites. Asian J. Fundam. Appl. Sci..

[53] Kwon H., Leparoux M., Heintz J.M., Silvain J.F., Kawasaki A. (2011). Fabrication of single crystalline diamond reinforced aluminum matrix composite by powder metallurgy route. Met. Mater. Int..

[54] Yang X., Zou T., Shi C., Liu E., He C., Zhao N. (2016). Effect of carbon nanotube (CNT) content on the properties of in-situ synthesis CNT reinforced Al composites. Mater. Sci. Eng. A.

[55] Amore S., Ricci E., Borzone G., Novakovic R. (2008). Wetting behaviour of lead-free Sn-based alloys on Cu and Ni substrates. Mater. Sci. Eng. A.

[56] Lasky R.C. Copper-Tin intermetallics: Their importance, growth rate, and nature. Proceedings of the SMTA International 2018 Conference Brochure.

[57] Jiang M.G., Xu C., Nakata T., Yan H., Chen R.S., Kamado S. (2016). Development of dilute Mg-Zn-Ca-Mn alloy with high performance via extrusion. J. Alloys Compd..

[58] Mota J.M., Martinez M.A., Velasco F., Criado A.J. (2004). Preparation of aluminium boride by powder technology. Ceram. Int..

[59] Kayikci R., Kurtulus O., Gurbuz R. (2009). The formation and growth behavior of aluminium boride crystals in an Al-B alloy. Solid State Phenom..

[60] Ebhota W.S., Jen T.-C. (2018). Intermetallics Formation and Their Effect on Mechanical Properties of Al-Si-X Alloys. Intermet. Compd.-Form. Appl..

[61] Zhang Y., Wang X., Jiang S., Wu J. (2010). Thermo-physical properties of Ti-coated diamond/Al composites prepared by pressure infiltration. Mater. Sci. Forum.

[62] Al-Dabbagh J.B., Tahar R.M., Ishak M., Harun S.A. (2015). Structural and phase formation of TiAl alloys synthesized by mechanical alloying and heat treatment. Int. J. Nanoelectron. Mater..

[63] Pal U., Sandoval A., Madrid S.I.U., Corro G., Sharma V., Mohanty P. (2016). Mixed titanium, silicon, and aluminum oxide nanostructures as novel adsorbent for removal of rhodamine 6G and methylene blue as cationic dyes from aqueous solution. Chemosphere.

[64] Jamal N.A., Tan A.W., Yusof F., Katsuyoshi K., Hisashi I., Singh S., Anuar H. (2016). Fabrication and compressive properties of low to medium porosity closed-cell porous Aluminum using PMMA space holder technique. Materials.

[65] Linul E., Marsavina L., Ková J. (2017). Collapse mechanisms of metal foam matrix composites under static and dynamic loading conditions. Mater. Sci. Eng. A.

[66] Salur E., Nazik C., Acarer M., Şavklıyıldız İ., Akdoğan E.K. (2021). Ultrahigh hardness in Y2O3 dispersed ferrous multicomponent nanocomposites. Mater. Today Commun..

[67] Aldoshan A., Khanna S. (2017). Effect of relative density on the dynamic compressive behavior of carbon nanotube reinforced aluminum foam. Mater. Sci. Eng. A.

